# Analysis of Potential Iron Toxicity in Hemodialysis Patients Under Intravenous Iron Treatment

**DOI:** 10.3390/medsci14010154

**Published:** 2026-03-21

**Authors:** Jessy Korina Peña-Esparragoza, Alina Chávez-Guillén, Paloma Ramos-López, Oscar Rueda-Elías, Susana López-Ongil, Matilde Alique, Rafael Ramírez-Chamond, Julia Carracedo, Diego Rodríguez-Puyol, Patricia Martínez-Miguel

**Affiliations:** 1 Nephrology Section, Príncipe de Asturias University Hospital, 28805 Alcalá de Henares, Spain; korinapenae@gmail.com (J.K.P.-E.); alina_c10@hotmail.com (A.C.-G.); drodriguez.hupa@gmail.com (D.R.-P.); 2 Radiodiagnostic Department, Santa Cristina University Hospital, 28009 Madrid, Spain; ramoslopezpaloma@hotmail.com; 3 Radiodiagnostic Department, Príncipe de Asturias University Hospital, 28805 Alcalá de Henares, Spain; oscarruedarx@yahoo.es; 4 Research Unit of the Biomedical Research Foundation, Principe de Asturias University Hospital, 28805 Alcalá de Henares, Spain; slorgil@salud.madrid.org; 5 Systems Biology Department, University of Alcalá, 28805 Alcalá de Henares, Spain; matilde.alique@uah.es (M.A.); manuel.ramirez@uah.es (R.R.-C.); 6 Instituto Ramón y Cajal de Investigación Sanitaria (IRYCIS), 28034 Madrid, Spain; 7 Instituto de Investigación Sanitaria Hospital 12 de Octubre (IMAS12), 28041 Madrid, Spain; julcar01@ucm.es; 8 Genetics, Physiology and Microbiology Department, Faculty of Biological Sciences, Complutense University, 28040 Madrid, Spain; 9 Medicine Department, University of Alcalá, 28805 Alcalá de Henares, Spain

**Keywords:** iron toxicity, anemia, hemodialysis

## Abstract

**Background/Objectives**: Higher iron doses are used in the anemia treatment of hemodialysis patients, which allows for lower doses of erythropoiesis-stimulating agents; however, there are concerns regarding the risk of iron toxicity. This study aimed to evaluate the potential toxicity of iron deposition in prevalent hemodialysis patients on iron therapy and its relationship with parameters used to assess iron status, plasma protein oxidation, and cellular iron toxicity. **Methods**: Magnetic resonance imaging was performed in 56 patients to assess hepatic iron deposition, which was related to clinical and analytical parameters. In patients included in the first and fourth quartiles, according to hepatic iron deposition, plasma protein oxidative stress was quantified, as were iron and cytokine levels in peripheral blood mononuclear cells (PBMCs). **Results**: Patients with higher hepatic iron deposition had a longer time on hemodialysis (42.0 ± 43.0 vs. 4.9 ± 3.4 months, *p* < 0.001) and higher ferritin levels (1200 ± 516 vs. 429 ± 278 ng/mL, *p* < 0.001) than those with lower hepatic iron deposition, without differences in transferrin saturation or hepatic enzyme serum concentration. No differences were found in plasma protein oxidation, iron content, or cytokine mRNA content in PBMCs, except for a decrease in IL-6 levels in patients with higher hepatic iron deposition. **Conclusions**: Patients with longer hemodialysis times had higher iron stores, suggesting that iron treatment over time increases hepatic iron deposition. No parameters supporting increased toxicity in patients with higher hepatic iron deposition were observed.

## 1. Introduction

In patients receiving maintenance hemodialysis, intravenous iron has become a standard treatment for the management of anemia [1]. Iron is an essential element in several metabolic routes, with particular significance as a heme group compound, and iron supplementation is regularly used even without severe iron deficiency in order to decrease exposure to erythropoiesis-stimulating agents [2,3].

The parameters currently available to assess iron deficiency in hemodialysis patients are mainly serum transferrin saturation (TSAT) and serum ferritin. In chronic kidney disease, absolute iron deficiency is defined as ferritin < 100 µ/L and TSAT less than 20% in patients not on hemodialysis therapy and as ferritin < 200 µ/L in hemodialysis patients. Relative or functional iron deficiency is the existence of adequate (or elevated) iron stores that cannot, however, be mobilized for erythrocytosis, and is extremely common in inflammatory environments. Although there are no homogeneous diagnostic criteria, clinical guidelines define relative iron deficiency as ferritin > 200 µ/L and a TSAT < 20% in hemodialysis patients [4,5,6]. Additionally, it is well known that anemia improvement is more effective as the iron dose is increased; however, there are concerns regarding its potential toxicity [7]. Iron is an essential element for the development of infectious microorganisms, and it is believed that excess iron may increase the risk of infections [8,9]. In addition, iron is involved in oxidation–reduction reactions, which can lead to oxidative stress. This may be a contributing factor to increased cardiovascular risk and damage to tissues and organs [10,11]. In fact, retrospective observational studies suggest an increased risk of infections and mortality with high-dose iron administration [12], and this is particularly relevant when iron stores are high; therefore, the KDIGO Anemia 2012 guideline proposes that iron treatment be avoided when ferritin is above 500 µg/L [2]. However, a recent prospective clinical trial has shown that there is no difference in the incidence of mortality, non-fatal cardiovascular events, and infections between patients treated with proactive (264 mg/monthly) or reactive (145 mg/monthly) doses of iron, considering a limit for ferritin levels higher than those recommended by the KDIGO guidelines (700 μg/L). In this previous study, patients treated with higher iron doses required fewer doses of erythropoiesis-stimulating agents and fewer blood transfusions [13]. Additionally, another recent meta-analysis failed to demonstrate a higher risk of infections or cardiovascular events in patients treated with intravenous iron during dialysis [14]. Therefore, there is controversy regarding the maximum dose of intravenous iron that can be used in hemodialysis patients and the levels of ferritin that can be assumed to avoid toxicity.

For this purpose, we aimed to evaluate the potential toxicity of iron deposition in prevalent hemodialysis patients on intravenous iron therapy and its relationship with the parameters used to assess iron status (ferritin and TSAT). In this study, we selected liver and peripheral blood mononuclear cells (PBMCs) to simultaneously test iron deposition and cell toxicity. We also evaluated plasma protein oxidation as a marker of increased oxidative stress.

## 2. Materials and Methods

### 2.1. Study Design and Population

This cross-sectional study included 56 patients with end-stage renal disease. Patients were those aged over 18 years, undergoing in-center thrice-weekly hemodialysis for at least six months, and who were treated with intravenous iron sucrose. Patients with documented liver disease or cancer were excluded. None of the patients had a previous renal transplant. Moreover, the prevalence of hypertension, diabetes, primary cause of kidney failure, and vascular access were registered. This study was conducted in accordance with the principles of the Declaration of Helsinki and was approved by the Medicine Research Ethics Committee of Principe de Asturias University Hospital (code number: KPPM-Hx). Written informed consent was obtained from all patients included in the study. Baseline and demographic characteristics of the patients were obtained from the medical records, and the data recorded were anonymized.

All patients were dialyzed in the same center. Water and dialysate were evaluated monthly according to the “Guidelines for Quality Management of Dialysis Fluid” of the Spanish Society of Nephrology [15]. In hemodialysis, purified water was used with the European and Spanish Pharmacopoeia Recommendations. The bacterial counts of standard purified water and endotoxins were less than 100 CFU/mL and 0.25 EU/mL, respectively, with a maximum conductivity of 5 µS/cm at 25 °C.

Magnetic resonance imaging (MRI) was performed in all patients. In the same month as the MRI, pre-dialysis blood samples were collected in the middle of the week for the analyses detailed below. The mean weekly doses of intravenous iron sucrose, erythropoiesis-stimulating agents, and the active form of vitamin D in the last 3 months were quantified. The patients included were not being treated with immunosuppressive drugs.

### 2.2. Magnetic Resonance Imaging

MRI evaluates, in an intense magnetic field, the exponential decrease in liver signal after a stimulation wave. The decrease depends on T1 and T2 relaxation times. Iron atoms have a paramagnetic effect inducing a T1 and T2 decrease in the nearby hydrogen nuclei, and their grouping in clusters of ferritin or hemosiderin crystals increases this effect, which becomes superparamagnetic, leading mainly to a reduction in the T2 of the adjacent protons in the spin-echo sequence. The Rennes University Protocol provides scientifically validated quantification of iron and hepatic fat from gradient echo sequences [16,17]. The MRI signal intensity was measured in five regions of approximately1 cm^2^: three were measured in the liver parenchyma, excluding vascular structures, and one was measured in each paraspinal muscle. This process was repeated for the five MRI sequences, and these values were used to compute five different liver-to-muscle signal intensity ratios, which were then analyzed using the algorithm developed by Gandon et al. and provided on the website of the University of Rennes [16]. According to the Rennes scale, a normal iron deposit is considered to be less than 40 mosm/g; a mild iron overload, between 41 and 100 mosm/g; a moderate iron overload, between 101 and 200 mosm/g; and a severe iron overload, >200 mosm/g. Image analysis was blinded, and interobserver variability was assessed using the interclass correlation coefficient, with a concordance of 0.92. Hepatic fat fraction was accounted for in iron quantification.

### 2.3. Laboratory Analysis

#### 2.3.1. Evaluation of Liver Function, Anemia, Iron Metabolism-Related Parameters, and Inflammatory Status

Routine blood tests were performed, including measurements of hemoglobin, serum ferritin, transferrin, iron, transaminase, alkaline phosphatase, and C-reactive protein. Serum levels of alkaline phosphate, aspartate aminotransferase, alanine aminotransferase, gamma-glutamyl transferase, iron, and C-reactive protein were determined using a colorimetric method with an Atellica^®^ analyzer (Siemens Healthineers, Tarrytown, NY, USA), while blood hemoglobin levels were determined using a dual method with a hematology autoanalyzer (Advia 2120i Hematology Systems; Siemens Healthineers, Erlangen, Germany). Moreover, ferritin was measured via chemiluminescence, and transferrin via immunoturbidimetry using an Atellica^®^ Analyzer (Siemens Healthineers, Tarrytown, NY, USA).

#### 2.3.2. Quantification of Iron Content in PBMCs

An iron assay kit (ab83366, ABCAM, Cambridge, MA, USA) was used to quantify the total Fe, Fe^2+^, and Fe^3+^ in the PBMCs. Cells (103/mL) were homogenized in an iron assay buffer on ice and subsequently centrifuged at 16,000× *g* to remove insoluble materials. The supernatants were then transferred to a clean tube and kept on ice. Thereafter, 25 µL of the sample was added to a 75 µL iron assay buffer and 5 µL iron reducer, which reduced Fe^3+^ to Fe^2+^. Next, 100 µL of the iron-probe solution was added to generate an Fe^2+^-ferrene S complex that absorbs light at 593 nm, and spectrophotometry was used to detect the absorbance at this wavelength. To normalize the results, the protein content of the samples was calculated and expressed as nmol Fe/mg protein.

#### 2.3.3. Evaluation of Proinflammatory Cytokine mRNA Content in PBMCs

Total RNA was extracted from PBMCs using the QIAcube standard protocol (RNeasy QIAcube Kit; QIAGEN, Hilden, Germany) according to the manufacturer’s instructions. Moreover, cDNA was synthesized using the High-Capacity cDNA Archive Kit (Applied Biosystems, Foster City, CA, USA), and 1 µg of total RNA primed with random hexamer primers, using a T100 PCR thermal cycler (Bio-Rad, Hercules, CA, USA), according to the manufacturer’s instructions. Real-time polymerase chain reaction (PCR) was performed using the ABI Prism 7500 sequence detection PCR system (TaqMan^®^ Universal Master Mix II, No AmpErase^®^ UNG; Applied Biosystems), according to the manufacturer’s protocol. Additionally, specific TaqMan assay probes from humans used CCL2 or monocyte chemoattractant protein-1 (MCP1) (Hs00234140_m1), tumor necrosis factor alpha (TNF-α) (Hs00174128_m1), interleukin 6 (IL-6) (Hs00174131_m1), and HPRT1(Hs02800695_m1; normalized assay). The mRNA copy numbers were calculated for each sample using the instrument’s software and a comparative threshold (Ct) value. After normalization to internal controls (HPRT1 expression levels), the relative fold-change was determined using the 2^−ΔΔCt^ method. Furthermore, gene fold expression was calculated from the difference between the gene expression under each experimental condition.

#### 2.3.4. Evaluation of Hydroxynonenal Protein Adducts in Plasma

Hydroxynonenal (HNE) protein adducts, an accepted marker of oxidative stress [18], were measured in the plasma using an OxiSelect HNE adduct competitive ELISA Kit (STA-838) from Cell Biolabs, Inc. (San Diego, CA, USA). This ELISA kit is an enzyme immunoassay developed for the rapid detection and quantitation of HNE protein adducts. The ELISA plate was coated with HNE conjugate, and unknown HNE protein samples from plasma or HNE-BSA standards were added to the HNE-conjugate preabsorbed ELISA plate. After a brief incubation period of 10 min at R/T, an anti-HNE polyclonal antibody was added for 1 h at R/T, followed by an HRP-conjugated secondary antibody for 1 h at R/T. The content of HNE protein adducts in unknown samples was determined by comparison with a predetermined HNE-BSA standard curve after reading the absorbance at a wavelength of 450 nm, following the manufacturer’s instructions.

### 2.4. Statistical Analysis

Categorical variables are presented as frequencies and percentages, and continuous variables are presented as the mean ± standard deviation or median (interquartile range). Categorical variables were compared using the Chi-square test. The distribution of continuous variables was verified using the Shapiro–Wilk normality test. A non-parametric analysis was performed using the Kruskal–Wallis test to evaluate the differences between the different groups and the Dunn-Bonferroni test in the post hoc analysis for pair comparisons. A multiple linear regression test was performed with variables with statistical significance over iron hepatic deposition, and Pearson’s correlation coefficient was used to analyze the association between ferritin levels and hepatic iron deposition. The non-parametric Mann–Whitney U test was used to compare quartiles. All analyses were conducted using the statistical software IBM^®^ SPSS^®^ Statistics 20, and statistical significance was defined as *p*-value < 0.05.

## 3. Results

Hepatic iron deposition was measured on MRI in 56 patients. These patients were divided into four categories, from normal to severe hepatic iron deposition, according to the Rennes score. The baseline patient characteristics are shown in Table 1. Table 2 shows hemodialysis-related data according to the liver iron deposition classified using the Rennes score. Most patients presented with slight iron deposition (41–100 mosm/g), and patients with higher hepatic iron deposition had a longer time on hemodialysis and higher serum ferritin levels. With the multiple linear regression analysis (model significance: *p* < 0.001, R^2^ = 0.56), both ferritin (β = 0.62, *p* < 0.001) and time on hemodialysis (β = 0.23, *p* = 0.025) maintained their statistical significance over hepatic iron deposition.

The association between hepatic iron deposition assessed on MRI and ferritin was significant (r = 0.72, *p* < 0.001). No statistically significant differences were observed in the other parameters compared, including liver enzyme activity or markers of systemic inflammation such as C-reactive protein.

**Figure 1:**
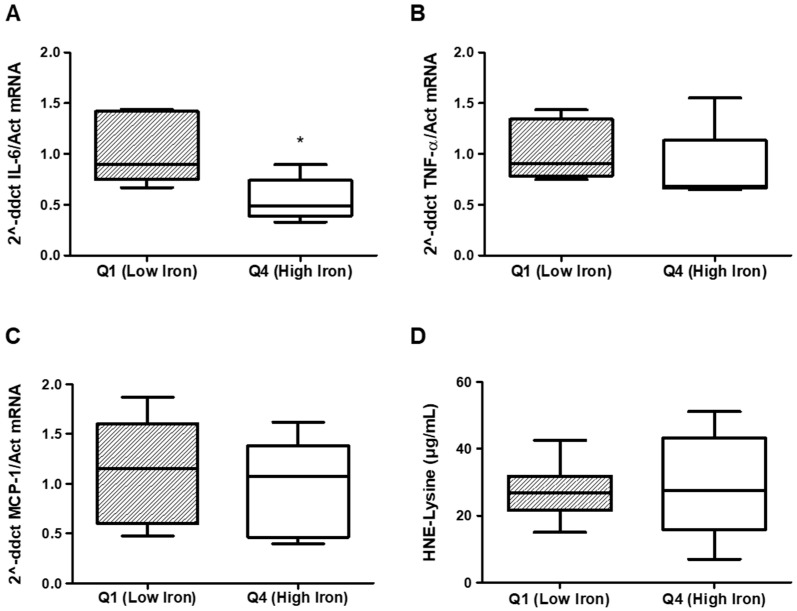
Proinflammatory cytokine mRNA content in peripheral blood mononuclear cells (PBMCs) and hydroxynonenal (HNE) protein adducts in plasma in patients in the first (Q1) and fourth (Q4) quartiles, according to iron overload stratified by Rennes score. IL-6: interleukin 6; TNF-α: tumor necrosis factor alpha; MCP-1: monocyte chemoattractant protein-1; HNE-Lysine: 4-hydroxynonenal- lysine. * *p* < 0.05. Regarding the inflammatory parameters assessed in PBMCs, no differences were found for TNF-α and MCP-1 (**B**,**C**), but lower IL-6 levels were detected in patients with higher hepatic iron deposition (**A**). No differences were found in the adducted plasma content of the HNE protein (**D**).

Additionally, we performed a more focused analysis in patients from the two extremes—the first and fourth quartiles—of the hepatic iron deposition distribution quantified on MRI. The mean iron deposition was 30.3 ± 9.3 mosm/g and 185.1 ± 49.5 mosm/g in the first and fourth quartiles, respectively (Table 3). In this analysis, patients with higher iron deposition upon MRI were on hemodialysis for a longer time; however, there was no difference in the mean weekly dose of intravenous iron therapy and erythropoiesis-stimulating agents in the last 3 months (Table 3). These patients also had higher ferritin levels, with no differences in liver toxicity parameters, and C-reactive protein levels between the groups (Table 4). TSAT levels were similar in the first and fourth quartiles according to hepatic iron deposition measured on the Rennes scale (Table 4). Moreover, no differences were found between these two groups with regard to PBMC iron content (Table 5) or the plasma content of HNE protein adducts (Figure 1). Regarding the inflammatory parameters assessed in PBMCs, no differences were found for MCP-1 and TNF-α; however, lower IL-6 levels were detected in patients with higher hepatic iron deposition (Figure 1).

## 4. Discussion

Intravenous iron therapy is useful for improving anemia in hemodialysis patients. Increasing the total amount of iron administered can reduce the requirement for erythropoiesis-stimulating agents [13]. However, there are concerns regarding the potential toxicity of iron overload. The KDIGO 2012 Anemia guideline recommends the administration of intravenous iron with TSAT levels ≤ 30% and ferritin ≤ 500µ/L [2]. However, these recommendations are mainly based on observational studies, and the safe upper limits for TSAT and ferritin remain unknown [4].

Considering the available information, we have some doubts regarding the relevance of ferritin concentrations in prescribing iron therapy. Ferritin is not a good parameter for assessing iron availability for erythropoiesis, and hemoglobin and TSAT levels alone may be more appropriate for deciding how to administer intravenous iron. Moreover, we question the evidence that the iron requisitioned in the liver or circulating immune cells of hemodialysis patients could induce significant tissue damage or facilitate infection by pathogenic bacteria, since it is not free in blood circulation. Iron is a transition metal that is highly active in oxidation–reduction reactions, and it must be bound to a ligand to avoid damage to cells and tissues. These ligands mainly include transferrin, heme, and ferritin. Iron not bound to these ligands, called non-transferrin-bound iron (NTBI), appears to be responsible for damage both on cell surfaces and intracellularly [19,20] and may be a risk factor for Gram-negative and other siderophilic bacteria [8,9].

In this study, we evaluated the potential toxicity of iron deposition in prevalent hemodialysis patients receiving intravenous iron therapy, as well as its relationship with the parameters used to assess iron status (ferritin and TSAT). We found that patients with higher hepatic iron overload were those with a longer time on hemodialysis; however, they did not differ in the current mean weekly iron and erythropoiesis-stimulating agent therapy, suggesting that the differences found could be better explained by a higher cumulative dose of iron over time. In fact, if we administer the same weekly dose of iv iron for a longer period of time, with a sustained erythropoiesis rate, and without clinically relevant blood loss, an iron-positive balance will occur in a time-dependent fashion. Similarly, patients with higher iron deposition had higher ferritin levels in the absence of significant differences in TSAT, supporting the opinion that ferritin is a good marker of hepatic iron deposition. In our opinion, this does not mean that elevated ferritin levels, associated with high iron deposits, cause toxicity. However, ferritin is also considered an acute phase reactant, so hemodialysis patients may have elevated ferritin levels for other reasons. In this study, the association between hepatic iron deposition assessed on MRI and ferritin was significant (r = 0.72, *p* < 0.001); however, when considering this value, it seems that ferritin levels may not be completely explained just for the intracellular iron concentration-mediated regulation of the ferritin gene. Other previously proposed factors may be involved, such as uremia-dependent inflammation, dialysis technique, or vascular access. In this study, these factors were controlled by measuring C reactive protein and urea serum levels, vascular access, and dialysis technique, without detecting differences among the different groups.

Regarding iron deposition in organs other than the liver, such as the heart or pancreas, MRI studies have shown that iron deposition has a different pattern and is not correlated with serum ferritin levels [21,22,23,24,25]. Our results support these findings, as no differences were observed in the iron content of PBMCs between the two experimental groups tested. Therefore, our results indicate that only TSAT values agree with the KDIGO recommendations, and this is due to doubts regarding the clinical value of ferritin as a good marker for anemia treatment in hemodialysis patients.

**Figure 2:**
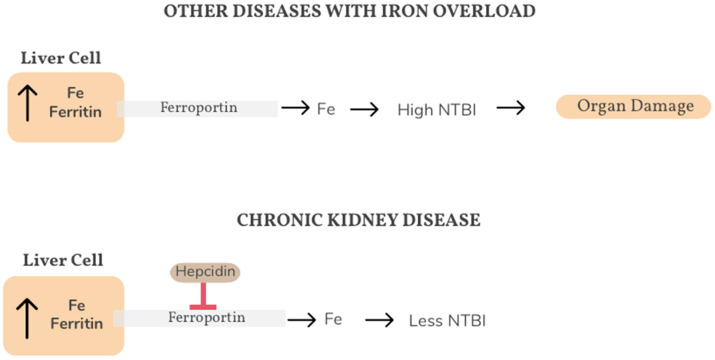
Organ damage associated with non-transferrin-bound iron (NTBI). In diseases with iron overload, both hepatic and circulating iron levels are increased, leading to an elevation of circulating NTBI. This NTBI seems to be responsible for organ damage as it induces oxidative stress and lipid peroxidation. In patients with chronic kidney disease, different circumstances converge to increase hepcidin production. Hepcidin regulates overall iron balance by causing the breakdown of iron transporter ferroportin, which decreases the flow of iron into the blood from reserves in the body, decreasing circulating NTBI.

Regarding hepatic toxicity, we were unable to detect significant changes in the parameters generally used to assess liver function, such as the circulating levels of hepatic enzymes, an accepted marker of liver damage. These findings differ from previously published evidence concerning other diseases characterized by iron liver accumulation. For example, in patients with untreated homozygous hemochromatosis, chronic liver disease is a feature of the clinical course of the disease. In these patients, both hepatic and circulating iron levels are increased, leading to an elevation of circulating NTBI [19]. This NTBI seems to be responsible for organ damage in hemochromatosis, as it induces oxidative stress, and lipid peroxidation [25]. In contrast with hemochromatosis, in patients with chronic kidney disease, different circumstances converge to increase hepcidin production in liver cells [4]. Hepcidin blocks the activity of ferroportin, a transmembrane protein that allows the efflux of iron from cells to plasma, promoting the iron sequestration within hepatic cells [26]. Consequently, circulating NTBI would not increase in the plasma of iron-overloaded hemodialysis patients and liver toxicity would not develop (shown in Figure 2).

Our results do not directly support this hypothesis, as we did not measure NTBI in our patients; therefore, the proposed mechanism is speculative and was not directly demonstrated in this cohort. However, plasma HNE protein adduct concentrations are considered a rather good index of systemic oxidative stress, and they did not increase in patients with higher Rennes scores. Consequently, it does not appear probable that patients with hepatic iron overload exhibit higher plasma concentrations of oxidative stress inducers, including NTBI.

High TSAT and ferritin levels are well known analytical markers for hemochromatosis. Thus, it would be possible that some of the patients included in our study could be carriers of this gene, since the prevalence of heterozygous carriers in hemodialysis patients is similar to that of the general population, around 6% [27]. However, we did not perform a genetic evaluation on the patients, as they did not meet the two conditions that we consider may constitute an indication for hemochromatosis gene screening: high TSAT and ferritin levels prior to iron treatment or an increase in these same parameters in the absence of a historical record of iron status.

Other studies have revealed a high prevalence of severe hepatic iron overload in patients with end-stage renal disease treated with hemodialysis, considering this a deleterious effect that may aggravate or trigger nonalcoholic fatty liver disease [28,29,30,31]. However, most of these studies did not carefully analyze liver function, and a recent one [31] showed that hepatic iron load increased the accumulation of the fat fraction in the liver of dialysis patients but not the plasma concentration of liver enzymes. The O’Lone meta-analysis [28] compares the deleterious effects of oral versus intravenous iron administration. The authors report that there is insufficient evidence to determine whether intravenous iron therapy is justified by its benefits on anemia and point to potential adverse effects such as hepatic iron overload. However, they conclude that longer follow-up periods are needed to determine this. Consequently, although the general opinion of the scientific community is that iron accumulation in the liver is toxic, we believe that there is no definitive evidence demonstrating liver toxicity through analytical or histological data, or a worse clinical outcome in relation to liver disease in dialysis patients with hepatic iron overload.

Concerning another suggested toxic effect of iron overload, the potential increase in infections, we decided to evaluate some aspects of the behavior of circulating mononuclear cells. As previously stated, no changes were observed in either intracellular iron concentrations or mRNA expression of the inflammatory cytokines MCP-1 and TNF-α, in patients with high and low hepatic iron deposition and ferritin levels. These findings support the absence of relevant effects of iron administration in these cell subpopulations, but they do not exclude an effect on polymorph nuclear cells or tissue macrophages. Surprisingly, IL-6 mRNA expression decreased in PBMCs of patients with increased hepatic iron deposition. These changes do not seem to be related to changes in the intracellular iron of the PBMCs, as neither ferric nor ferrous iron concentrations differed between the groups tested. The implication of this finding is unclear, as IL-6 is a pro-inflammatory cytokine. However, it plays an important role in the immune response; therefore, its deficiency could potentially affect it. Previous studies analyzing the relationship between iron and IL-6 are scarce. Nakagawa and cols. demonstrated an inverse correlation between serum IL-6 levels and iron serum concentration [32], an association that could be related to our finding of decreased IL-6 mRNA expression in PBMCs of patients with hepatic iron overload. However, additional studies are needed to confirm the consistency of this finding and, especially, to explain the pathological relevance of this relationship, since due to the small sample size of this study, these results should be considered exploratory and not definitive. In addition, the absence of statistically significant differences in some biomarkers should be interpreted with caution, as a type II error cannot be excluded. However, the values for MCP-1 and HNE-Lysine are very similar in both groups, and perhaps only in the case of TNF-α measurements could differences possibly be detected if the sample size were increased, although the results would be consistent with those obtained for IL-6.

Our study had some limitations. First, NTBI was not measured and PBMC iron levels do not necessarily reflect iron accumulation in Kupffer cells, tissue macrophages, cardiac tissue, or the labile plasma iron pool. Another limitation is that, although our results strongly suggest a cumulative dose of iron over time on hemodialysis, this cumulative dose was not quantified. In addition, this was a cross-sectional study; therefore, causality could not be demonstrated. Furthermore, we are not sure either whether high hepatic iron stores could have negative consequences for patients in the future, since no follow-up was performed. However, studies on the long-term consequences of liver damage in hemodialysis patients, such as the development of cirrhosis or hepatocarcinoma, have shown associations with virus infection [33], but not with iron overload. In addition, the number of patients included in this study was limited. Although a previous randomized clinical trial has shown that intravenous iron therapy in hemodialysis patients with a high ferritin limit of 700 μg /L [13] is safe, further studies are necessary to confirm the safety at higher-dose intravenous iron and higher ferritin levels.

## 5. Conclusions

In summary, in hemodialysis patients treated with intravenous iron and ferritin levels higher than those recommended by clinical practice guidelines, our results do not support the appearance of increased liver toxicity or proinflammatory and oxidative damage. Therefore, we believe that intravenous iron therapy for anemia treatment may not necessarily induce toxicity in hemodialysis patients and that the available markers have limitations in determining the risk of toxicity. Our results suggest that only TSAT values agree with the KDIGO recommendations, and although further confirmatory studies are needed, ferritin levels above guideline-recommended values are likely to be safe. In the future, we may have new tools for assessing iron toxicity, such as the NTBI, which may facilitate treatment decisions [20]. Additionally, new treatments involving hepcidin to improve iron bioavailability, such as inhibitors of hypoxia-inducible factor and prolyl hydroxylase or monoclonal antibodies targeting the hepcidin–ferroportin pathway, are currently being developed [34,35].

## Figures and Tables

**Table 1:** Characteristics of the patients at baseline according to liver iron deposition classified by Rennes scor.

Characteristic		Liver Iron Quantification (mosm/g)			
	Normal (≤40) N = 12	Slight (41–100) N = 30	Moderate (101–200) N = 8	Severe (>200) N = 6	*p*
Age, mean ± SD, years	64.8 ± 23.4	63.9 ± 15.7	62.9 ± 16.2	59.2 ± 15.9	0.50
Male, n (%)	8 (66.7)	26 (86.7)	6 (75.0)	3 (50.0)	0.20
Diabetes, n (%)	10 (83.3)	19 (63.3)	6 (75.0)	2 (33.3)	0.18
Hypertension, n (%)	10 (83.3)	27 (90.0)	7 (87.5)	4 (83.3)	0.93
Primary cause of kidney failure, n (%)					0.81
hypertension	0 (0)	5 (16.7)	1 (12.5)	2 (33.3)	
diabetic nephropathy	5 (41.7)	7 (23.3)	3 (37.5)	1 (16.7.0)	
hypertension	2 (16.7)	3 (10.0)	1 (12.5)	0 (0)	
glomerular disease	3 (25.0)	8 (26.7)	1 (12.5)	1 (16.7.0)	
inherited kidney conditions	0 (0.0)	2 (6.7)	0 (0)	1 (16.7)	
others	2 (16.7)	5 (16.3)	2 (25)	1 (16.7)	
Hemodialysis technique, n (%)					0.90
on-line hemodiafiltration.	7 (58.3)	18 (60)	5 (62.5)	3 (50)	
Conventional high-flux hemodialysis	5 (41.7)	12 (40)	3 (37.5)	3 (50)	
Duration of dialysis treatment, mean ± SD, months	4.2 ± 1.5	11.8 ± 15.9	29.8 ± 26.6 *	59.2 ± 57.7 *	0.02
Vascular access, n (%)					0.87
arteriovenous fistula	7 (58.3)	19 (63.3)	4 (50.0)	3 (50.0)	
catheter	5(41.7)	11 (36.7)	4 (50.0)	3 (50.0)	
Hemoglobin, mean ± SD, g/dL	11.3 ± 1.4	11.4 ± 1.1	11.4 ± 0.7	11.7 ± 0.6	0.90
Ferritin, mean ± SD, ng/mL	425 ± 260	561 ± 329	906 ± 364	1591 ± 431 **	<0.001
Transferrin saturation, mean ± SD, %	26.1 ± 8.0	26.6 ± 6.6	27.6 ± 8.5	32.3± 9.9	0.40
C- reactive protein level, mean ± SD, mg/L	19.8 ± 20.5	18.7 ± 19.9	11.9 ± 9.0	31.1 ± 21.3	0.34
Aspartate aminotransferase, mean ± SD, UI/L	19.2 ± 7.1	18.1 ± 4.6	18.9 ± 6.3	21.4 ± 8.7	0.70
Alanine aminotransferase, mean ± SD, UI/L	14.8 ± 5.4	17.0 ± 5.6	19.5 ± 7.3	15.7 ± 10.1	0.30
Gamma-glutamyl transferase, mean ± SD, UI/L	79.8 ± 134.2	52.7 ± 44.5	43.6 ± 34.7	34.0 ± 25.8	0.55
Alkaline phosphate, mean ± SD, UI/L	120.0 ± 94.6	100.2 ± 46.4	104.8 ± 37.0	90.1 ± 20.3	0.85
Erythropoiesis-stimulating agent dose, mean ± SD,µg/week	45.0 ± 31.9	40.2 ± 37.5	20.6 ± 11.0	51.7 ± 32.9	0.16
Intravenous iron dose, mean ± SD, mg/week	65.1 ± 27.0	98.4 ± 79.4	72.8 ± 79.8	118.8 ± 44.7	0.09

* *p* < 0.05 with moderate and severe versus with normal liver iron quantification. ** *p* < 0.05 with severe versus with normal and slight liver iron quantification.

**Table 2:** Hemodialysis-related data according to liver iron deposition classified by Rennes score.

Characteristic		Liver Iron Quantification (mosm/g)			
	Normal (≤40) N = 12	Slight (41–100) N = 30	Moderate (101–200) N = 8	Severe (>200) N = 6	*p*
Albumin, mean ± SD, mg/dL	3.6 ± 0.5	3.7 ± 0.4	3.9 ± 0.4	3.6 ± 0.3	0.36
Total protein, mean ± SD, mg/dL	6.4 ± 0.5	6.4 ± 0.5	6.3 ± 0.4	6.3 ± 0.6	0.94
Sodium, mean ± SD, mEq/L	138 ± 2	139 ± 3	137 ± 2	140 ± 2	0.21
Potassium, mean ± SD, mEq/L	4.8 ± 0.4	5.0 ± 0.6	5.1 ± 0.5	4.9 ± 0.6	0.33
Calcium, mean ± SD, mg/dL	8.9 ± 0.4	8.7 ± 0.5	9.0 ± 0.6	8.6 ± 0.5	0.50
Phosphorus, mean ± SD, mg/dL	4.0 ± 0.8	4.5 ± 0.8	4.4 ± 0.4	4.2 ± 0.8	0.30
Bicarbonate, mean ± SD, mEq/L	21.5 ± 3.4	21.1 ± 2.2	21.2 ± 1	21.9 ± 0.8	0.90
Serum urea, mean ± SD, mg/dL	124 ± 29	115 ± 37	136 ± 24	105 ± 31	0.31
Intact PTH (i-PTH), mean ± SD, pg/mL	377 ± 168	453 ± 221	592 ± 235	428 ± 361	0.23
Active Vitamin D doses, mean ± SD, mcg/week	3.4 ± 1.9	3.3 ± 2.6	3.3 ± 3	3.2 ± 1.8	0.34
Kt/V	1.4 ± 0.1	1.4 ± 0.2	1.5 ± 0.12	1.6 ± 0.2	0.67

Kt/V: K: dialyzer clearance of urea; t: dialysis time; V: volume of distribution of urea; SD: standard deviation.

**Table 3:** Some baseline characteristics in patients in the first (Q1) and fourth (Q4) quartiles, according to iron overload stratified by Rennes score.

Characteristic	Liver Iron Deposition		
	Q1 (Low Iron) N = 14	Q4 (High Iron) N = 14	*p*-Value
Fe MRI, mean ± SD, mosm/g	30.3 ± 9.3	185.1 ± 49.5	<0.001
Age, mean ± SD	67.3 ± 22.6	61.3 ±15.2	0.42
Male, n (%)	9 (64.3)	9 (64.3)	1.00
Albumin, mean ± SD, mg/dL	3.6 ± 0.5	3.7 ± 0.4	0.50
Total protein, mean ± SD, mg/dL	6.5 ± 0.5	6.3 ± 0.5	0.39
Sodium, mean ± SD, mEq/L	138 ± 2	138 ± 2	0.50
Potassium, mean ± SD, mEq/L	4.8 ± 0.5	5.0 ± 0.6	0.27
Calcium, mean ± SD, mg/dL	8.8 ± 0.4	8.8 ± 0.6	0.94
Phosphorus, mean ± SD, mg/dL	4.0 ± 0.8	4.3 ± 0.6	0.18
Bicarbonate, mean ± SD, mEq/L	21.5 ± 3.2	21.5 ± 0.9	0.99
Serum urea, mean ± SD, mg/dL	124± 38	123 ± 31	0.94
Intact PTH (i-PTH), mean ± SD, pg/mL	379 ± 172	517 ± 280	0.13
Active Vitamin D doses, mean ± SD, mcg/week	3.9 ± 2.5	3.3 ± 2.4	0.50
Kt/V	1.4 ± 0.1	1.4 ± 0.1	0.20
Vascular access, N (%)			0.70
Arteriovenous fistula	8 (57.1)	7 (50.0)	
Catheter	6 (42.9)	7 (50.0)	
Duration of dialysis treatment, mean ± SD, months	4.9 ± 3.5	42.4 ± 43.0	0.01
Erythropoiesis-stimulating agent therapy, mean ± SD, (µg/week)	44.9 ± 30.8	34.0 ± 27.1	0.33
Intravenous iron therapy, mean ± SD, (mg/week)	62.9 ± 32.1	92.5 ± 69.0	0.16

Fe: iron; MRI: magnetic resonance imaging; SD: standard deviation; Kt/V: K: dialyzer clearance of urea; t: dialysis time; V: volume of distribution of urea.

**Table 4:** Iron status, systemic inflammation, and liver cell function in patients in the first (Q1) and fourth (Q4) quartiles, according to iron overload stratified by Rennes score.

Characteristic	Liver Iron Deposition		
	Q1 (Low Iron) N = 14	Q4 (High Iron) N = 14	*p*-Value
Hemoglobin, mean ± SD, g/dL	11.3 ± 1.3	11.5 ± 0.7	0.48
Ferritin, mean ± SD, ng/mL	429 ± 278	1200 ± 516	<0.001
Transferrin saturation, mean ± SD, %	26. 4 ± 8.8	29.6 ± 9.0	0.36
C-reactive protein level, mean ± SD, mg/L	19.6 ± 20.0	20.2 ± 17.8	0.93
Aspartate aminotransferase, mean ± SD, UI/L	18.9 ± 7.1	20.0 ± 7.2	0.88
Alanine aminotransferase, mean ± SD, UI/L	15.3 ± 6.1	17.9 ± 6.1	0.36
Gamma-glutamyl transferase, mean ± SD, UI/L	75.8 ± 124.5	39.5 ± 30.5	0.31
Alkaline phosphate, mean ± SD, UI/L	112.8 ± 90.1	98.5 ± 30.9	0.58

SD: standard deviation.

**Table 5:** Quantification of iron content in peripheral blood mononuclear cells in patients in the first (Q1) and fourth (Q4) quartiles, according to iron overload stratified by Rennes score.

Characteristic	Liver Iron Deposition		
	Q1 (Low Iron) N = 14	Q4 (High Iron) N = 14	*p*-Value
Total iron, median (interquartile range) (nmol/mg protein)	0.48 (0.77)	1.03 (0.87)	0.24
Ferrous iron (Fe^2+^), median (interquartile range) (nmolFe/mg protein)	0.30 (0.45)	0.47 (0.45)	0.18
Ferric iron (Fe^3+^), median (interquartile range) (nmolFe/mg protein)	0.21 (0.74)	0.38 (0.50)	0.14

## Data Availability

The original contributions presented in this study are included in the article. Further inquiries can be directed to the corresponding author.

## Abbreviations

The following abbreviations are used in this manuscript:

PBMCs	Peripheral blood mononuclear cells
TSAT	Serum transferrin saturation
MRI	Magnetic resonance imaging
HNE	Hydroxynonenal
NTBI	Non-transferrin-bound iron
